# In Vitro Activity of Novel Lipopeptides against Triazole-Resistant *Aspergillus fumigatus*

**DOI:** 10.3390/jof8080872

**Published:** 2022-08-18

**Authors:** Simona Fioriti, Oscar Cirioni, Oriana Simonetti, Lucia Franca, Bianca Candelaresi, Francesco Pallotta, Damian Neubauer, Elzbieta Kamysz, Wojciech Kamysz, Benedetta Canovari, Lucia Brescini, Gianluca Morroni, Francesco Barchiesi

**Affiliations:** 1 Department of Biomedical Sciences and Public Health, Polytechnic University of Marche, 60126 Ancona, Italy; 2 Infectious Disease Clinic, Azienda Ospedaliero Universitaria “Ospedali Riuniti”, 60126 Ancona, Italy; 3 Dermatological Unit, Department of Clinical and Molecular Sciences, Polytechnic University of Marche, 60126 Ancona, Italy; 4 Infectious Diseases Unit, Azienda Ospedaliera Ospedali Riuniti Marche Nord, 61122 Pesaro, Italy; 5 Department of Inorganic Chemistry, Faculty of Pharmacy, Medical University of Gdańsk, 80-210 Gdańsk, Poland; 6 Laboratory of Chemistry of Biological Macromolecules, Department of Molecular Biotechnology, Faculty of Chemistry, University of Gdańsk, 80-309 Gdańsk, Poland

**Keywords:** *Aspergillus fumigatus*, azole resistance, antimicrobial peptides, lipopeptides

## Abstract

Aspergillosis, which is mainly sustained by *Aspergillus fumigatus*, includes a broad spectrum of diseases. They are usually severe in patients with co-morbidities. The first-line therapy includes triazoles, for which an increasing incidence of drug resistance has been lately described. As a consequence of this, the need for new and alternative antifungal molecules is absolutely necessary. As peptides represent promising antimicrobial molecules, two lipopeptides (C14-NleRR-NH_2_, C14-WRR-NH_2_) were tested to assess the antifungal activity against azole-resistant *A. fumigatus*. Antifungal activity was evaluated by determination of minimum inhibitory concentrations (MICs), time–kill curves, XTT assay, optical microscopy, and checkerboard combination with isavuconazole. Both lipopeptides showed antifungal activity, with MICs ranging from 8 mg/L to 16 mg/L, and a dose-dependent effect was confirmed by both time–kill curves and XTT assays. Microscopy showed that hyphae growth was hampered at concentrations equal to or higher than MICs. The rising antifungal resistance highlights the usefulness of novel compounds to treat severe fungal infections. Although further studies assessing the activity of lipopeptides are necessary, these molecules could be effective antifungal alternatives that overcome the current resistances.

## 1. Introduction

The rates of Aspergillosis, a broad spectrum of illness that includes noninvasive and invasive forms, are currently rising every year [1,2,3]. The invasive forms are usually associated with recognized risks factors such as neutropenia and hematologic malignancies, allogeneic bone marrow transplantation, solid organ transplant (SOT), neoplasm, or HIV patients. Furthermore, the current pandemic of SARS-CoV-2 led to an increase in Invasive Pulmonary Aspergillosis (IPA) [4]. The first-line treatment for Aspergillosis included triazoles (voriconazole and isavuconazole) and liposomal amphotericin B as alternative therapy. In some cases, echinocandins, posaconazole, and itraconazole were also used, with the last two drugs administered for prophylaxis against IPA [1]. However, in the last years the azole resistance in *Aspergillus* spp., especially in *A. fumigatus* (the most frequent species involved in Aspergillosis [1]), increased, with rates reaching 3.2% globally but with high regional differences [5]. Italian data showed that azole-resistant clinical/environmental *Aspergillus* spp. represented 7% of the isolates [6]. The resistance mechanisms included alteration in the *cyp51A* gene and promoter and upregulation of its expression [5]. In addition to the prolonged administration of the azoles in clinical settings, the increased use of these antifungals in agriculture also contributed to the diffusion of resistance [7]. The increasing resistance to drugs prompted the search for unconventional strategies to overcome infections sustained by resistant isolates. One of the promising approaches is the use of antimicrobial peptides (AMPs). AMPs or cationic host defense peptides are small peptides of 50 or less amino acids with antibacterial properties and immunomodulatory activity. Since their discovery in the 1960s, several studies described different molecules, their potential as antimicrobial agents, and their action aiming at both intracellular targets and membranes [8]. AMPs are also versatile instruments: the numerous structures could be used as bases to design and develop new analogues with enhanced activities [8]. Although the main field of application of these molecules regarded the activity against bacteria and viruses, the AMPs also retained activity against fungi, including filamentous species and molds [9,10,11]. 

The aim of the present work was to assess the antifungal activity of two antimicrobial lipopeptides (C14-NleRR-NH_2_, C14-WRR-NH_2_) against *A. fumigatus* and evaluate a possible interaction of these peptides with isavuconazole. 

## 2. Materials and Methods

### 2.1. Isolates

Two azole-resistant strains of *A. fumigatus*, namely SSI-4524 and SSI-5586, harboring TR34/L98H and G54W mutations of the Cyp51A, respectively, were used. *Candida krusei* ATCC 6258 was used as quality control for antifungal susceptibility testing.

### 2.2. Antifungals

Isavuconazole (Merck, Milano, Italy) was diluted in DMSO. 

### 2.3. Peptide Synthesis

The compounds (C14-NleRR-NH_2_, C14-WRR-NH_2_) were obtained by using the method reported previously [12]. These peptides were developed in a previous work assessing the impact of ultrashort cationic lipopeptides on both antimicrobial and hemolytic activities and chosen due to their antibacterial activity [12]. Briefly, lipopeptides were synthesized manually on Rink Amide resin by solid-phase Fmoc/tBu methodology. Deprotection of the Fmoc group was carried out with a 20% (*v*/*v*) piperidine solution in N,N-dimethylformamide (DMF) for 15 min. Acylation was performed with an equimolar mixture of N,N’-diisopropylcarbodiimide, OxymaPure, and Fmoc-AA-OH dissolved in DMF and dichloromethane (1:1, *v*/*v*) in fourfold excess based on the resin for 1.5 h. Fmoc-L-Arg(Pbf)-OH, Fmoc-L-Nle-OH, Fmoc-L-Trp(Boc)-OH, and tetradecanoic acid were used in coupling reactions. The peptide C14-NleRR-NH_2_ was cleaved from the resin using a mixture (A) of TFA, TIS, and deionized water (95:2.5:2.5, *v*/*v*/*v*), and C14-WRR-NH_2_ using a mixture (B) of TFA, phenol, TIS, and deionized water (92.5:2.5:2.5:2.5, *v*/*v*/*v*/*v*). Cleavage was accomplished within 1.5 h under stirring. The peptides were purified by RP-HPLC (>95%, HPLC) and lyophilized. The identity of all compounds was confirmed by mass spectrometry (ESI–MS). C14-NleRR-NH_2_ and C14-WRR-NH_2_ were diluted in water and stock solutions were stored at 4 °C. For use, the stocks were diluted to working solution in RPMI 1640 medium (Merck, Milano, Italy).

### 2.4. Preparation of Fungal Suspensions

*A. fumigatus* isolates were grown on Sabouraud agar plates (Oxoid, Wesel, Germany) for 2–5 days at 35 °C. Isolates were resuspended in a solution of water plus 0.1% tween 20. The solution was filtered using a filter membrane of 11 µm pore size (Merck, Milano, Italy) for the recovery of the conidia. The suspension was diluted to obtain a final solution at a concentration of 2–5 × 10^5^.

### 2.5. MIC Determination

To determine the minimal inhibitory concentration (MIC) of C14-NleRR-NH_2_, C14-WRR-NH_2_, and isavuconazole, broth microdilution was performed following the EUCAST methodology [13]. Briefly, 1–2 × 10^5^ CFU/mL was seeded in RPMI-1640 plus scalar concentrations of drugs (from 0.125 to 128 mg/L) and incubated at 35 °C.

### 2.6. Time–Kill Assay

Growth curves were performed to evaluate the action of the peptides. Fungal growth was monitored measuring OD_450_ nm every hour for 24 h. Briefly, 2–5 × 10^5^ CFU/mL was seeded in RPMI-1640 and incubated at 35 °C. For both peptides, the concentrations used were 0.5X, 1X, and 2X MIC. Untreated isolates were used as control. 

### 2.7. XTT Assay

XTT assay was performed to evaluate the action of the peptides. XTT assays were performed using 0.5X, 1X, and 2X MIC of both peptides. A positive control without peptides was also performed. The fungal inoculum (1–2 × 10^5^ CFU/mL) was incubated in flat-bottomed 96-well plates at 35 °C. XTT reduction was measured after 0 h, 8 h, and 24 h. Two hours prior to the end of incubation time, 50 µL of 2.5 mg/mL XTT (Life Technologies, Monza, Italy) plus 125 µM menadione (Merck, Milano, Italy) solutions were added to each well. A total of 150 µL of the suspensions was transferred to new 96-well plates with a flat bottom. The absorbance for each well was read at 492 nm with a multiplate reader [14].

### 2.8. Optical Microscopy

The isolates were grown on flat-bottomed 96-well plates in the presence of RPMI alone and with different concentrations of peptides. At established times (0 h, 8 h, and 24 h), the observation was performed using an inverted microscope (Axiovert 25, Zeiss, Roma, Italy) with 100× magnification.

### 2.9. Checkerboard Assays

The combination between peptides and isavuconazole was assessed with the checkerboard method (following the EUCAST recommendations for media, inoculum, antifungal dilutions, and result reading), interpreting the interactions obtained with the classification suggested by Odds [15].

### 2.10. Statistical Analysis

All experiments were performed in triplicates. All graphs were made using GraphPad Prism 7. The level of statistical significance was determined using the Dunnett’s multiple comparison test, *p* < 0.05 was stated as significant, and the confidence score was indicated by asterisks: *: *p* < 0.05.

## 3. Results

The antifungal and peptide MICs for the isolates are reported in Table 1. *A. fumigatus* SSI-4524 showed MIC values for isavuconazole, C14-NleRR-NH2, and C14-WRR-NH2 of 8 µg/mL. *A. fumigatus* SSI-5586 exhibited an MIC value of 16 µg/mL for both peptides and 1 µg/mL for isavuconazole. 

### 3.1. Time–Kill Assay

**Figure 1:**
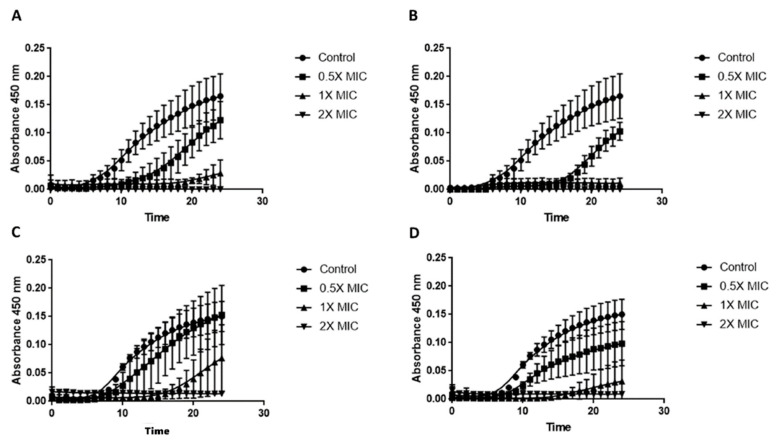
Time–kill curves of two *A. fumigatus* isolates. *A. fumigatus* SSI-5586 treated with different concentrations of C14-NleRR-NH_2_ (**A**) and C14-WRR-NH_2_ (**B**). *A. fumigatus* SSI-4524 treated with different concentrations of C14-NleRR-NH_2_ (**C**) and C14-WRR-NH_2_ (**D**).

Figure 1 shows the growth curves of the two *A. fumigatus* isolates treated with different concentrations of peptides. Both peptides already had a marked inhibitory activity against planktonic *A. fumigatus* cells after 9 h of incubation. For *A. fumigatus* SSI-5586, the differences between control and treated growth curves for both peptides became statistically significant after 9 h. While 1X and 2X MIC concentrations resulted in the absence of growth, the addition of 0.5X MIC of C14-NleRR-NH_2_ and C14-WRR-NH_2_ delayed the start of growth by 4 and 10 h compared to control, respectively (Figure 1A,B). The results obtained with *A. fumigatus* SSI-4524 were slightly different. Although the treatment with 2X MIC of both peptides hampered the growth of the isolates, the 0.5X and 1X MIC treatments were less active compared to *A. fumigatus* SSI-5586. Indeed, the use of 0.5X MIC of C14-NleRR-NH_2_ did not result in a statistically different curve compared to the control. Moreover, C14-WRR-NH_2_ at 0.5X MIC, perhaps reducing the growth, delayed the start of the growth by only 2 h compared to control (Figure 1C,D). 

### 3.2. XTT Assay

**Figure 2:**
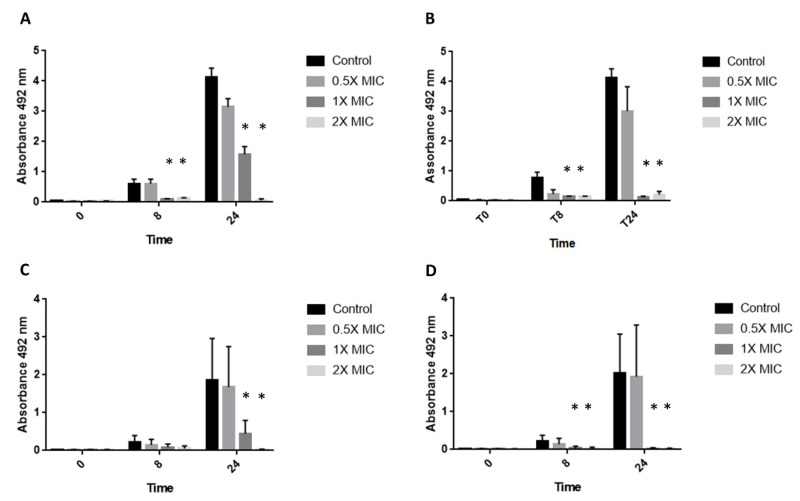
XTT assay of two isolates of *A. fumigatus*. *A. fumigatus* SSI-5586 treated with different concentrations of C14- NleRR-NH_2_ (**A**) and C14-WRR-NH_2_ (**B**). *A. fumigatus* SSI-4524 treated with different concentrations of C14- NleRR-NH_2_ (**C**) and C14-WRR-NH_2_ (**D**). *: *p* < 0.05 compared to control.

The XTT assay results were consistent with those obtained from the time–kill curves. Treatment of the isolate SSI-5586 with 1X and 2X MIC concentrations of the two peptides already resulted in a significant difference in absorbance compared to the control at 8 h. Consistent with the results obtained in time–kill curves, 0.5X MIC concentrations of both peptides did not inhibit the fungal growth, resulting in a reduction in XXT after 24 h of incubation (Figure 2A,B). The *A. fumigatus* SSI-4524-only treatment with 1X MIC and 2X MIC concentrations for 24 h resulted in a significant difference, while SUB-MIC concentrations of the peptides were not effective in inhibiting the metabolic activity (Figure 2C,D).

### 3.3. Optical Microscopy

**Figure 3:**
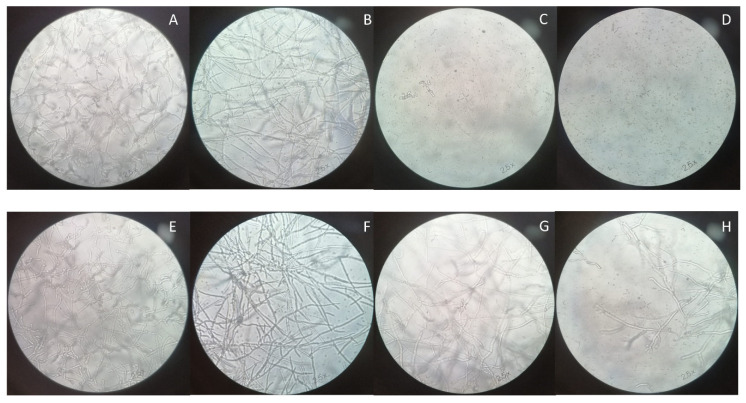
Optical microscopy of the *A. fumigatus* isolates after 24 h of treatment with C14-NleRR-NH_2_ at different concentrations. *A. fumigatus* SSI-4524 untreated (**A**), treated with 0.5X MIC (**B**), 1X MIC (**C**), and 2X MIC (**D**). *A. fumigatus* SSI-5586 untreated (**E**), treated with 0.5X MIC (**F**), 1X MIC (**G**), and 2X MIC (**H**).

**Figure 4:**
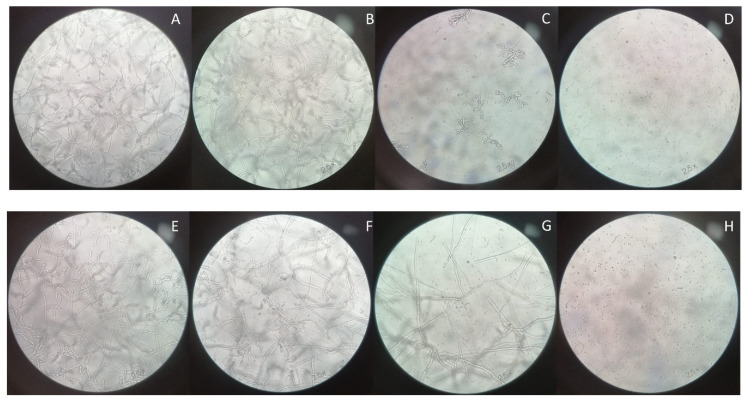
Optical microscopy of the *A. fumigatus* isolates after 24 h of treatment with C14-WRR-NH_2_ at different concentrations. *A. fumigatus* SSI-4524 untreated (**A**), treated with 0.5X MIC (**B**), 1X MIC (**C**), and 2X MIC (**D**). *A. fumigatus* SSI-5586 untreated (**E**), treated with 0.5X MIC (**F**), 1X MIC (**G**), and 2X MIC (**H**).

Microscopy observations after 24 h of incubation with the two peptides are shown in Figure 3 and Figure 4. Both peptides interfered with the mycelial growth in a concentration-dependent manner. Indeed, while 0.5X MIC concentrations produced a limited reduction of the hyphae, more pronounced effects were detected at 1X MIC and 2X MIC. In the latter case, the fungal growth was completely inhibited, according to the results obtained with time–kill and XTT assays. 

### 3.4. Checkerboard Assays

To evaluate the activity of the peptides in combination with isavuconazole, checkerboard assays were performed. The combination yielded indifferent interaction.

## 4. Discussion

Aspergillus infections are continuously rising and have become a cause of concern in immunocompromised patients or in concomitance with other diseases. Voriconazole and itraconazole represent the first-line therapy, while amphotericin B and echinocandins are considered in azole-resistant strains [16]. Alongside the increasing incidence and the challenging diagnosis of Aspergillosis, resistance to azoles is a cause for concern. Moreover, the use of azoles in agriculture raises the selection of resistance isolates in the environment and threatens the clinical efficacy of antifungal drugs [17].

The low number of therapeutic options and the high mortality of these infections have prompted the search for new antifungals, and peptides represent a class of natural molecules with promising characteristics to develop new drugs. Even though antimicrobial peptides were deeply studied for their antibacterial and immunomodulatory activities, the antifungal activity of these compounds was evaluated in several works. A number of molecules demonstrated killing activity against various species of yeast, mainly *Candida* [9]. 

In addition to peptides, lipopeptides seem to be very promising molecules with broad activity against bacteria and fungi [18,19,20,21]. Nevertheless, the few peptide-derived drugs approved for clinical use belong to the lipopeptide class or its derivatives, e.g., dalbavacin is a semisynthetic glycopeptide approved for acute bacterial skin and soft tissue infections [22], while daptomycin is a cyclic lipopeptide used for the treatment of Gram-positive infections [23]. Interestingly, these drugs demonstrate wound healing properties in addition to antibacterial activities [23,24,25]. Similar to antibiotics, some antifungal drugs also belong to the lipopeptide class: echinocandins are cyclic lipopeptides with fungicidal activity against most pathogenic yeast but with limited efficacy against filamentous fungi, and in particular *Aspergillus* spp. [26]. Despite this, echinocandins could have a role in the treatment of *Aspergillus* and in particular of invasive aspergillosis, and new formulations could enhance the activity of this class of antifungals. Moreover, other lipopeptides with different chemical formulations have proven efficacy against *Aspergillus* spp. Makovitzki and colleagues synthesized different lipopeptides that demonstrated activity against bacteria, yeast, and *Aspergillus*: they also revealed that although the lipopeptides’ activity against microorganisms was mainly due to the membrane disruption, some molecules acted via different mechanisms [27], suggesting that different formulations could enhance the activity and increase the spectrum. 

Antimicrobial activities of C14-NleRR-NH_2_ and C14-WRR-NH_2_ were previously tested against *Staphylococcus aureus, Pseudomonas aeruginosa*, and *Candida albicans*, and both lipopeptides also showed activity against ESKAPE pathogens [12]. Our results demonstrated an additional antifungal activity against *Aspergillus*, highlighting the broad activity of lipopeptides and their derivatives. Both molecules had a similar activity, with stackable MICs and comparable microscopical effects. Time–kill and XTT assays denoted a slightly higher activity of C14-WRR-NH_2_, but a reduction in viable cells and metabolic activity was achieved with the 1X MIC concentration. Optical observation instead demonstrated that both peptides hampered the mycelial growth of *A. fumigatus* SSI-4524 at the 1X MIC concentration, while for *A. fumigatus* SSI-5586 the reduction in hyphae was remarkable with the 2X MIC concentration. These behaviors could be related to strain-specific properties, and further studies are needed to understand the mechanisms of susceptibility to these novel peptides.

Few studies reported the activity of AMPs against *Aspergillus* spp. Despite some antimicrobial host defense peptides demonstrating good activity against *Aspergillus* [28], other studies revealed that well-studied AMPs, such as omiganan or temporin G, did not achieve significant results against filamentous fungi [29,30]. Conversely, lipopeptides seem to be more effective against *Aspergillus*: in a recent work Dell’Olmo and collaborators identified peptides derived from apolipoprotein B capable of reducing the metabolic activity of *Aspergillus niger* [31]. Similarly, Zhang et al., synthetized novel Lipo-γ-AA peptides with a broad spectrum of activity against both *Candida* and *Aspergillus* [32]. Moreover, one of these Lipo-γ-AA peptides (namely MW5) demonstrated a synergic activity when used in combination with fluconazole against *Candida*. In our experiments, the combination with isavuconazole did not show synergistic effects, but these results could be due to the different chemical structures of the peptides and to the different species tested. Although there are few data concerning the combination of peptides and antifungals, some work revealed the synergic activities of peptide and fluconazole combinations in *Candida* species [32]. Since we tested just isavuconazole, we cannot exclude that other antifungal drugs could synergize with our peptides: further studies including novel strains and different antifungal drugs should be performed to reveal possible interactions useful in clinical settings. 

The Identification of novel compounds with activity against *Aspergillus* is promising, taking into account the increasing rates of azole resistance in these species. The use of triazole in clinical settings and in agriculture increased the incidence of azole-resistant isolates and is gaining attention due to the possible clinical failure [33]. The resistance mechanisms to triazoles involve mutation and overexpression of the *cyp51* gene, efflux pumps, and other mechanisms [33]. AMPs instead act in two different ways: they provoke a membrane perturbation, with subsequent lysis of the membrane, or enter the cells through endocytosis [9]. The different targets allow the use of lipopeptides against azole-resistant *Aspergillus*: our results demonstrated that both C14-NleRR-NH_2_ and C14-WRR-NH_2_ are effective against the two resistant isolates tested, and this further prompts the research of lipopeptides as alternatives in antifungal therapy. 

## 5. Conclusions

The rising problem of antimicrobial resistance is a cause of concern that requires quick responses and novel approaches. AMPs, and in particular lipopeptides, are versatile molecules also possessing antifungal properties and could be promising alternatives to the current antifungal therapy. Although they deserve further studies to reveal their actual potential, our in vitro results demonstrated their great activity against this difficult-to-treat opportunistic pathogen. 

## Figures and Tables

**Table 1:** Antifungal susceptibility profiles of isolates used.

Strains	MIC (µg/mL)		
	C14-NleRR-NH2	C14-WRR-NH2	Isavuconazole
*A. fumigatus* SSI-4524	8	8	8
*A. fumigatus* SSI-5586	16	16	1

## Data Availability

The data presented in this study are available on request from the corresponding author.
